# Exploring Deep Physiological Models for Nociceptive Pain Recognition

**DOI:** 10.3390/s19204503

**Published:** 2019-10-17

**Authors:** Patrick Thiam, Peter Bellmann, Hans A. Kestler, Friedhelm Schwenker

**Affiliations:** 1 Institute of Medical Systems Biology, Ulm University, Albert-Einstein-Allee 11, 89081 Ulm, Germany; hans.kestler@uni-ulm.de; 2 Institute of Neural Information Processing, Ulm University, James-Franck-Ring, 89081 Ulm, Germany; peter.bellmann@uni-ulm.de (P.B.); friedhelm.schwenker@uni-ulm.de (F.S.)

**Keywords:** convolutional neural networks, signal processing, information fusion, pain intensity classification

## Abstract

Standard feature engineering involves manually designing measurable descriptors based on some expert knowledge in the domain of application, followed by the selection of the best performing set of designed features for the subsequent optimisation of an inference model. Several studies have shown that this whole manual process can be efficiently replaced by deep learning approaches which are characterised by the integration of feature engineering, feature selection and inference model optimisation into a single learning process. In the following work, deep learning architectures are designed for the assessment of measurable physiological channels in order to perform an accurate classification of different levels of artificially induced nociceptive pain. In contrast to previous works, which rely on carefully designed sets of hand-crafted features, the current work aims at building competitive pain intensity inference models through autonomous feature learning, based on deep neural networks. The assessment of the designed deep learning architectures is based on the *BioVid Heat Pain Database (Part A)* and experimental validation demonstrates that the proposed uni-modal architecture for the electrodermal activity (EDA) and the deep fusion approaches significantly outperform previous methods reported in the literature, with respective average performances of 84.57% and 84.40% for the binary classification experiment consisting of the discrimination between the baseline and the pain tolerance level ($T_{0}$ vs. $T_{4}$) in a *Leave-One-Subject-Out* (LOSO) cross-validation evaluation setting. Moreover, the experimental results clearly show the relevance of the proposed approaches, which also offer more flexibility in the case of transfer learning due to the modular nature of deep neural networks.

## 1. Introduction

Conventional machine learning approaches are built upon a set of carefully engineered representations, which consist of measurable parameters extracted from raw data. Based on some expert knowledge in the domain of application, a feature extractor is designed and used to extract relevant information in the form of a feature vector from the preprocessed raw data. This high level representation of the input data is subsequently used to optimise an inference model. Although such approaches have proven to be very effective and can potentially lead to state-of-the-art results (given that the set of extracted descriptors is suitable for the task at hand), the corresponding performance and generalisation capability is limited by the reliance on expert knowledge as well as the inability of the designed model to process raw data directly and to dynamically adapt to related new tasks.

Meanwhile, deep learning approaches [1] automatically generate suitable representations by applying a succession of simple and non-linear transformations on the raw data. A deep learning architecture consists of a hierarchical construct of several processing layers. Each processing layer is characterised by a set of parameters that are used to transform its input (which is the representation generated by the previous layer) into a new and more abstract representation. This specific hierarchical combination of several non-linear transformations enables deep learning architectures to learn very complex functions as well as abstract descriptive (or discriminative) representations directly from raw data [2]. Moreover, the hierarchical construct characterising deep learning architectures offers more flexibility when it comes to adapting such approaches to new and related tasks. Hence, deep learning approaches have been outperforming previous state-of-the-art machine learning approaches, especially in the field of image processing [3,4,5,6,7]. Similar performances have been achieved in the field of speech recognition [8,9] and natural language processing [10,11].

A steadily growing amount of work has been exploring the application of deep learning approaches on physiological signals. Martinéz et al. [12] were able to significantly outperform standard approaches built upon hand-crafted features by using a deep learning algorithm for affect modelling based on physiological signals (two physiological signals consisting of Skin Conductance (SC) and Blood Volume Pulse (BVP) were used in this specific work). The designed approach consisted of a multi-layer Convolutional Neural Network (CNN) [13] combined with a single-layer perceptron (SLP). The parameters of the CNN were trained in an unsupervised manner using denoising auto-encoders [14]. The SLP was subsequently trained in a supervised manner using backpropagation [15] to map the outputs of the CNN to the target affective states. In [16], the authors proposed a multiple-fusion-layer based ensemble classifier of stacked auto-encoder (MESAE) for emotion recognition based on physiological data. A physiological-data-driven approach was proposed in order to identify the structure of the ensemble. The architecture was able to significantly outperform the existing state-of-the-art performance. A deep CNN was also successfully applied in [17] for arousal and valence classification based on both electrocardiogram (ECG) and Galvanic Skin Response (GSR) signals. In [18], a hybrid approach using CNN and Long Short-Term Memory (LSTM) [19] Recurrent Neural Network (RNN) was designed to automatically extract and merge relevant information from several data streams stemming from different modalities (physiological signals, environmental and location data) for emotion classification. Moreover, deep learning approaches have been applied on electromyogram (EMG) signals for gesture recognition [20,21] or hand movement classification [22,23]. Most of the reported approaches consist of first transforming the processed EMG signal into a two dimensional (time-frequency) visual representation (such as a spectrogram or a scalogram) and subsequently using a deep CNN architecture to proceed with the classification. A similar procedure was used in [24] for the analysis of electroencephalogram (EEG) signals. These are just some examples of an increasingly growing field of experimentation for deep neural networks. A better overview of deep learning approaches applied to physiological signals can be found in [25,26]. However, there are few related works that focus specifically on the application of deep neural networks on physiological signals for pain recognition. The authors of [27] recently proposed a classification architecture based on Deep Belief Networks (DBNs) for the assessment of patients’ pain level during surgery, using photoplethysmography (PPG). The proposed architecture consists of a bagged ensemble of DBNs, built upon a set of manually engineered features, extracted from the recorded and preprocessed PPG signals. It is important to note that, in this specific study, the ensemble of bagged DBNs was trained on a set of carefully designed hand-crafted features. Therefore, an expert knowledge in this specific area of application is still needed in order to generate a set of relevant descriptors, since the whole classification process is not performed in an end-to-end manner.

Nonetheless, there is a constantly growing amount of works that focus specifically on pain recognition based on physiological signals, and categorised by the nature of the pain elicitations. There is a huge variety of statistical methods that have been proposed, most of them based on more traditional machine learning approaches such as decision trees or Support Vector Machines (SVMs) [28]. In [29], the authors proposed a continuous pain monitoring method using an Artificial Neural Network (ANN), based on hand-crafted features (wavelength (WL) and root mean square (RMS) features) extracted from several physiological signals consisting of heart rate (HR), breath rate (BR), galvanic skin response (GSR) and facial surface electromyogram (sEMG). The proposed approach was assessed on a dataset collected by inducing both thermal and electrical pain stimuli. In [30], the authors proposed a pain detection approach based on EEG signals. Relevant features are extracted from the EEG signals using the Choi–Williams quadratic time–frequency distribution and subsequently used to train a SVM in order to perform the classification task. Pain in this specific work is elicited throughout tonic cold. Most recently, Thiam et al. [31,32] provided the results for a row of pain intensity classification experiments based on the *SenseEmotion Database* (SEDB) [33], by using several fusion architectures to merge hand-crafted features extracted from different modalities, including physiological, audio and video channels. Thereby, the combination of the features extracted from the recorded signals was compared for different fusion approaches, namely the fusion at feature level, the fusion at the classifiers’ output level and the fusion at an intermediate level. Random Forests [34] were used as the base classifiers. In [35], the authors combined camera PPG input signals with ECG and EMG signals in order to proceed with a user-independent pain intensity classification using the same dataset. The authors used a fusion architecture at the feature level with Random Forests and SVMs as base classifiers.

In [36,37,38], the authors performed different pain intensity classification experiments based on the *BioVid Heat Pain Database* [39] (BVDB). All the conducted experiments were based on a carefully selected set of features extracted from both physiological and video channels. The classification was also performed using either Random Forests or SVMs. In [40], Kächele et al. performed a user-independent pain intensity classification evaluation based on physiological input signals, using the same dataset. The authors used the whole data from all recorded pain levels in a classification, as well as a regression setting with Random Forests as the base classifiers. Several personalisation techniques were designed and validated, based on meta information from the test subjects, distance measures and machine learning techniques. The same authors proposed an adaptive confidence learning approach for personalised pain estimation in [41] based on both physiological and video modalities. Thereby, the authors applied the fusion at feature level. The whole pain intensity estimation task was analysed as a regression problem. Random Forests were used as the base regression models. Moreover, a multi-layer perceptron (MLP) was applied to compute the confidence for an additional personalisation step. One recent work included the physiological signals of both datasets (SEDB and BVDB) [42]. The authors analysed different fusion approaches with fixed aggregating rules based on their merging level for the person-independent multi-class scenario using all available pain levels. Thereby, three of the most popular decision tree based classifier systems, i.e., Bagging [43], Boosting [44] and Random Forests, were compared.

The current work focuses on the application of deep learning approaches for nociceptive heat-induced pain recognition based on physiological signals (EMG, ECG and electrodermal activity (EDA)). Several deep learning architectures are proposed for the assessment of measurable physiological parameters in order to perform an end-to-end classification of different levels of artificially induced nociceptive pain. The current work aims at achieving state-of-the-art classification performances based on feature learning (the designed architecture autonomously extracts relevant features from the preprocessed raw signals in an end-to-end manner), therefore removing the reliance on expert knowledge for the design and optimisation of reliable pain intensity classification models (since most of the previous works on pain intensity classification involving autonomic parameters rely on a carefully designed set of hand-crafted features). The remainder of the work is organised as follows. The proposed deep learning approaches as well as the dataset used for the validation of the approaches are described in Section 2. Subsequently, a description of the results corresponding to the conducted assessments specific to each presented approach is provided in Section 3. Finally, the findings of the conducted experiments are discussed in Section 4, followed by the description of potential future works and a conclusion.

## 2. Materials and Methods

### 2.1. BioVid Heat Pain Database (BVDB)

The *BioVid Heat Pain Database* [39] (BVDB) was collected at Ulm University. It includes multi modal data recordings from healthy subjects subjected to different levels of artificially induced pain stimuli under strictly controlled conditions. The pain elicitation in the form of heat was conducted through the professionally designed PATHWAY (http://www.medoc-web/products/pathway) thermode attached to the participants’ right forearm. Before the data were recorded, a personalised calibration step was undertaken for each participant to determine individual levels for the pain threshold, as well as the tolerance threshold. Therefore, starting at a temperature of 32 °C (global pain free level $T_{0}$ for all participants), the temperature was slowly increased until, first, the participant felt a change from heat to pain (pain threshold $T_{1}$), and, second, the pain became hardly bearable (tolerance threshold $T_{4}$). In addition, two in-between pain elicitation levels $T_{2}$ and $T_{3}$ were calculated, making the four individual pain levels $T_{1},T_{2},T_{3},T_{4}$ equidistant. After the initial calibration steps, starting at the baseline temperature $T_{0}$, each of the four individual pain levels was applied randomly 20 times. Each of the pain levels was held for a total of 4 s. Each pain stimulation was followed by a rest period during which the baseline temperature was held for a random duration of 8–12 s. Ninety subjects were recruited for the experiments. The participants covered three age groups, i.e., 18–35 years, 36–50 years and 51–65 years. Each group was equally distributed, including 15 male and 15 female subjects. In the current study, the designed approaches were assessed on the *BioVid Heat Pain Database (Part A)* since most of the related works were conducted based on this specific database. The database is publicly available and consists of a total of 87 participants. Due to technical issues during the recordings, some of the data specific to three participants are missing [36]. Those participants were therefore discarded and the remaining 87 participants, for which all data are available, constitute the *BioVid Heat Pain Database (Part A)*.

**Figure 1:**
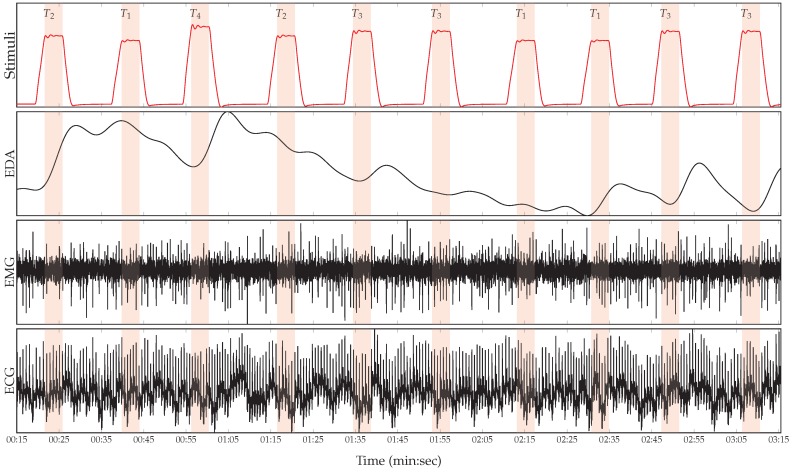
Recorded physiological data. From top to bottom: Series of artificially induced pain elicitation ($T_{1}$, pain threshold temperature; $T_{2}$, first intermediate elicitation temperature; $T_{3}$, second intermediate elicitation temperature; $T_{4}$, pain tolerance temperature); EDA (μS); EMG (μV); and ECG (μV).

During the experiments, three different physiological signals were recorded, namely electrodermal activity (EDA), electrocardiogram (ECG) and electromyogram (EMG) (a sample of the recorded physiological signals is depicted in Figure 1). The EDA is an indicator of the skin conductance level and was measured at both, the participants’ index and ring fingers. The ECG signals measure the participants’ heart activity, such as the heart rate, the interbeat interval and the heart rate variability. The EMG signal is an indicator of the muscle activity. The EMG signal of the current dataset consists of the muscle activities of the trapezius muscles, which are located at the back, in the shoulder area. In addition to the biopotentials, different video signals were recorded. Since in the current work we only consider the physiological signals, interested readers are referred to [39] to get further details on the whole dataset. Having 20 elicitations for each level of pain elicitation, every subject is represented by a total of 20×5=100 sequences of numerical data points (time series). Therefore, the unprocessed dataset consists of 87×100=8700 samples, each labelled with its corresponding level of nociceptive pain elicitation ($T_{0}$, $T_{1}$, $T_{2}$, $T_{3}$ or $T_{4}$).

### 2.2. Data Preprocessing

**Figure 2:**
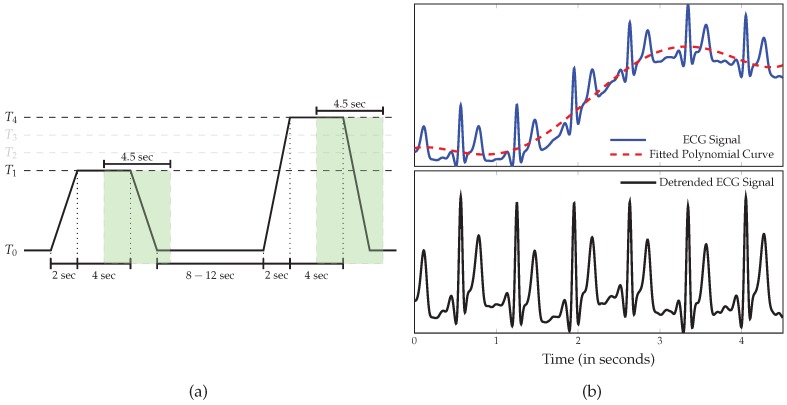
Data preprocessing. (**a**) Signal Segmentation. The classification experiments were performed on windows of length 4.5 s with a temporal shift of 4 s from the elicitations’ onset. (**b**) The ECG signal was further detrended by subtracting a least-squares polynomial fit from the preprocessed signal.

Prior to the classification experiments, the sampling rate of the recorded physiological modalities was reduced to 256 Hz, in order to reduce the computational requirements. Subsequently, the amount of noise and artefacts within the recorded data was significantly reduced by applying different signal preprocessing techniques on each specific modality. A third-order low-pass Butterworth filter with a cut-off frequency of 0.2 Hz was applied on the EDA signals. The EMG signals were filtered by applying a fourth-order bandpass Butterworth filter with a frequency range of $\left[20,250\right]$ Hz. Finally, a third-order bandpass Butterworth filter with a frequency range of $\left[0.1,250\right]$ Hz was applied on the ECG signals. Furthermore, the data were segmented as proposed in [37], but rather than using 5.5 s windows with a shift of 3 s from the elicitations’ onset, the preprocessed signals were segmented into windows of length 4.5 s, with a shift from the elicitations’ onset of 4 s (see Figure 2a), as recently proposed in [31]. Each signal extracted within this window constitutes a 1D array of size 4.5×256=1152 and was later used in combination with the corresponding level of nociceptive pain elicitation to optimise and assess the designed deep classification architectures. Thus, each physiological modality specific to each single participant is represented by a tensor with the dimensionality 100×1152×1. After some close analysis of the preprocessed physiological signals, a clear baseline wandering of the ECG signal, which is characterised by a strong correlation with the shape of the EDA signal, was observed (see Figure 2b). Therefore, the segmented ECG signals were additionally detrended by subtracting a fifth-degree polynomial least-squares fit from the filtered signals. This step was carried out to remove the aforementioned artefacts from the ECG signals, since these artefacts could potentially bias the classification performance of the corresponding deep classification model (instead of using information stemming uniquely from the ECG signal, the designed system would end up extracting information stemming from a non-linear combination of both the ECG signal and a noisy signal related to the EDA signal). Finally, data augmentation was performed by shifting the 4.5 s window of segmentation backward and forward in time with small shifts of length 250 ms and a maximal total window shift of 1 s in each direction, starting from the initial position of the window depicted in Figure 2a. The signals extracted within these windows were subsequently used as training material for the optimisation of the classification architectures.

### 2.3. Uni-modal Deep Model Description

As mentioned above, the goal of the current work is to apply feature learning to alleviate the reliance on domain specific expert knowledge that occurs when relevant and adequate features are to be manually designed (hand-crafted features) in order to achieve state-of-the-art classification performances. Therefore, multi-layer CNNs were designed and fed with the preprocessed physiological signals in order to automatically compute relevant signal representations and at the same time optimise the classification architectures. In the following sections, *c* depicts the number of classes of the classification task.

CNNs [45,46] constitute a distinct category of biologically inspired neural networks, which are characterised by a hierarchical structure of several processing layers. The input to a CNN is sequentially and progressively transformed by each specific layer and the back-propagated information stemming from the error computed between the network’s output and the expected output (ground-truth) is used to optimise the whole structure of the architecture in order to efficiently and effectively solve a classification or regression task. The basic processing layers of CNNs are *convolutional layers*, *pooling layers* and *fully connected layers*. *Convolutional layers* are characterised by a set of neurons (or kernels), whereby each specific neuron extracts a specific pattern of information from a patch of the layer’s input. Each neuron consists of a set of trainable weights, the size of which is determined by the patch’s size (or kernel size). The output of each neuron is calculated by applying a non-linear activation function (e.g., sigmoid function) on the weighted sum of the neuron’s input. Each neuron scans the layer’s input sequentially and the aggregation of the resulting local information extracted at each specific patch constitutes a feature map. Thus, the output of a convolutional layer is a set of feature maps generated by the convolution of each neuron across the layer’s input. *Pooling layers* reduce the spatial resolution of the generated feature maps by merging semantically similar features. *Max Pooling* is a commonly used pooling approach and consists of computing the maximum value of a defined local patch (the size of the patch related to a specific pooling layer is referred in the current work as “pool size”) of each feature map. *Fully connected layers* are basically single-layer feed-forward networks that perform the classification or regression task based on the learned deep representations.

Several challenges emerge when it comes to optimising such architectures. One of those challenges is the so-called *vanishing* or *exploding gradients* problem, which is caused by the *internal covariate shift* (constant fluctuations in layers’ input distributions) occurring in deep architectures during the training process. In [47], the authors proposed a technique called *Batch Normalisation* to address this specific issue. Batch Normalisation consists of automatically learning the optimal scaling and shifting parameters of each layer’s input, so that each layer’s input is dynamically normalised, thus significantly reducing the effects of the internal covariate shift and therefore stabilising the training process. Another common challenge occurring when training CNNs is the *overfitting* problem caused by the large amount of parameters that have to be consistently and effectively optimised. Applying regularisation techniques can help to significantly reduce this issue. The authors of [48] introduced the *dropout* approach, which is one of the most commonly used regularisation techniques for deep neural networks. The *dropout* approach consists of randomly and temporarily removing a set of neurons (or units) from the neural network during each training step, each neuron having a fixed probability $p\in \left[0,1\right]$ of being retained. The resulting model is therefore more robust against overfitting and generalises better.

In the current work, the designed architectures are regularised using both techniques and the dropout rate is fixed at 25%. Moreover, the *Exponential Linear Unit* (ELU) function [49] defined in Equation (1)

(1) $$\begin{array}{c} elu_{\alpha }\left(x\right)=\left\{\begin{array}{cc} \alpha \left(\exp(x)-1\right) & \mathrm{if}\;x<0\\ x & \mathrm{if}\;x\geq 0 \end{array}\right. \end{array}$$

is used as activation function for both convolutional layers and fully connected layers (with α=1), except for the last fully connected layer of each architecture where a *softmax* function defined in Equation (2)

(2) $$\begin{array}{c} s\left(y_{i}\right)=\frac{\exp(y_{i})}{\sum_{j}\exp\left(y_{j}\right)} \end{array}$$

is used as activation function, where $y_{i}=elu_{\alpha }\left(\sum_{k=1}^{n}w_{i,k}x_{k}+b_{i}\right)$ (${\left\{w_{i,k}\right\}}_{k=1}^{n}$ represents the set of weights of the *i*th neuron, $b_{i}$ represents the bias term of the *i*th neuron and $x=\left(x_{1},\ldots ,x_{k},\ldots ,x_{n}\right)$ represents the output of the precedent fully connected layer). The designed architectures for each physiological signal are based on 1D convolutional layers and are described in Table 1. The architectures are similar and were inspired by the architecture presented in [50] for the classification of ECG signals. The unique difference between the architectures is the usage of a dropout layer after each convolutional layer in the architecture specific to both modalities EMG and ECG.

### 2.4. Multi-Modal Deep Model Description

To further investigate the compatibility of the recorded physiological data, several fusion approaches based on CNNs are proposed. The information stemming from each modality is aggregated at different levels of abstraction.

**Figure 3:**
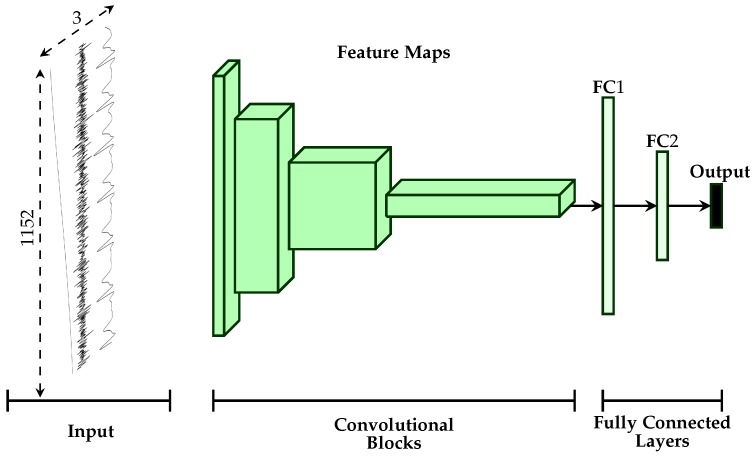
Early Fusion Architecture. A 2D representation of the input data is generated by concatenating the three physiological modalities and is subsequently fed into the designed deep architecture.

The first approach depicted in Figure 3 consists of an early fusion method, where the aggregation is done at the lowest level of abstraction, which consists of the preprocessed raw signals (input data). A 2D representation of the input data is generated by concatenating the three physiological modalities along the temporal axis, resulting in a tensor with the dimensionality 3×1152×1. The resulting data are subsequently fed into a network consisting of 2D convolutional layers. The motivation behind such an approach is to enable the architecture to dynamically learn an appropriate set of weights, which will generate feature maps consisting of relevant and compatible information extracted simultaneously from the recorded modalities, when applied to the 2D data representation. The designed fusion architecture is described in Table 2.

**Figure 4:**
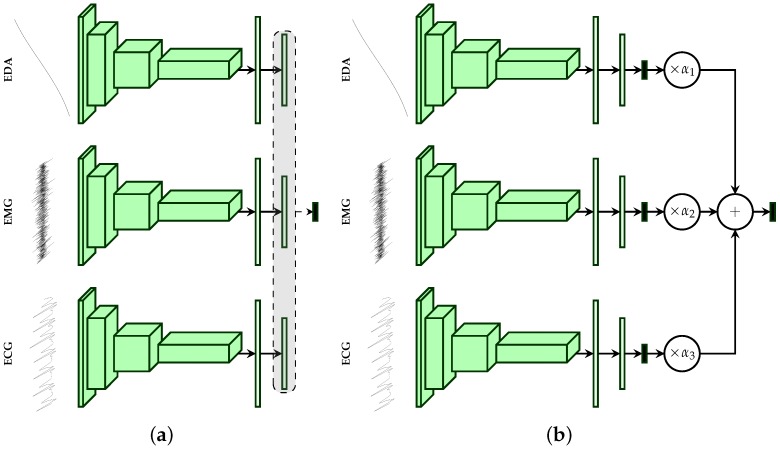
Late Fusion Architectures. (**a**) The features extracted by the second fully connected layer are concatenated and fed into the output layer. (**b**) The final output consists of a weighted average of the outputs of each uni-modal model.

Furthermore, two additional late fusion approaches are proposed (see Figure 4). Both approaches are based on the uni-modal CNN architectures described earlier (see Section 2.3). The first approach described in Figure 4a performs the aggregation of the information at the mid-level since it involves using intermediate representations of the input data. It consists of concatenating the outputs of the second fully connected layer of each modality specific architecture and feeding the resulting representation to an output layer with a *softmax* activation function. The second approach depicted in Figure 4b performs the aggregation at the highest level of abstraction, since it involves using the respective *softmax* layers’ outputs of each modality specific architecture. An additional layer consisting of a set of trainable positive parameters $\left(\alpha_{1},\alpha_{2},\alpha_{3}\right)\in R_{\geq 0}^{3}$ with a *linear* activation function is directly connected to the outputs of each uni-modal architecture.

For each modality specific architecture i∈{1,2,3} (since we are dealing with three physiological modalities), let $\left\{\theta_{i,j}\in \left[0,1\right]:1\leq j\leq c\right\}$ be the output values of the respective *softmax* layers. The output of the aggregation layer is computed by using the following formulas:

(3) $$\begin{array}{c} e_{j}=\frac{1}{3}\left(\sum_{i=1}^{3}\alpha_{i}\theta_{i,j}\right),\;\mathrm{with}\;\mathrm{the}\;\mathrm{constraint}:\;\sum_{i=1}^{3}\alpha_{i}=1 \end{array}$$

(4) $$\begin{array}{c} s\left(e_{j}\right)=e_{j} \end{array}$$

First, a weighted average output of the class probabilities stemming from the uni-modal architectures is computed (see Equation (3)), and the corresponding class probabilities of the fusion architecture are subsequently deducted by applying a *linear* activation function on the previously computed scores (see Equation (4)). Furthermore, the whole architecture is trained by using the loss function defined in Equation (5),

(5) $$\begin{array}{c} L=\sum_{i=1}^{3}\lambda_{i}L_{i}+\lambda_{agg}L_{agg} \end{array}$$

where $L_{1}$, $L_{2}$ and $L_{3}$ are the loss functions of each modality specific architecture and $L_{agg}$ is the loss function of the aggregation layer. The parameters $\lambda_{1}$, $\lambda_{2}$, $\lambda_{3}$ and $\lambda_{agg}$ are the corresponding weights for each of the loss functions. Once the architecture has been trained, unseen samples are classified based uniquely on the output of the aggregation layer. All described fusion approaches are subsequently trained in an *end-to-end* manner, which means that the fusion parameters are optimised at the same time as the parameters of each modality specific classification architecture. Furthermore, the parameters of each described architecture (uni-modal as well as multi-modal) are optimised using the cross entropy loss function defined in Equation (6),

(6) $$\begin{array}{c} \mathrm{Loss}=-\sum_{j=1}^{c}y_{j}\log\left({\hat{y}}_{j}\right) \end{array}$$

where $y_{j}$ is the ground-truth value of the *j*th class and ${\hat{y}}_{j}$ is the *j*th output value of the *softmax* function. Concerning the second late fusion architecture, the cross entropy loss function is used for each uni-modal architecture as well as for the aggregation layer ($L_{1}=L_{2}=L_{3}=L_{agg}=Loss$).

## 3. Results

All previously described deep architectures are trained using the Adaptive Moment estimation (*Adam*) [51] optimisation algorithm with a fixed learning rate set empirically to $10^{-5}$. The training process consisted of 100 epochs with the batch size set to 100. The weights of the loss function for the second late fusion architecture (see Figure 4b) were empirically set as follows: $\lambda_{1}=\lambda_{2}=\lambda_{3}=0.2$, $\lambda_{agg}=0.4$. The weight corresponding to the aggregation layer ($\lambda_{agg}$) was set higher than the others to push the network to focus on the weighted combination of the single modality architectures’ outputs, and therefore to evaluate an optimal set of the weighting parameters $\left\{\alpha_{1}\left(EDA\right),\alpha_{2}\left(EMG\right),\alpha_{3}\left(ECG\right)\right\}$. The implementation and evaluation of the described algorithms was done with the libraries Keras [52], Tensorflow [53] and Scikit-learn [54]. The evaluation of the architectures was performed in a *Leave-One-Subject-Out* (LOSO) cross-validation setting, which means that 87 experiments were conducted. During each experiment, the data specific to a single participant were used to evaluate the performance of the trained deep model and were never seen during the optimisation of this specific deep model. The data specific to each single participant were therefore used once as an unseen test set, and the results depicted in this section consist of averaged performance metrics from a set of 87 performance values.

A performance evaluation of the designed architectures in a binary classification task consisting of the discrimination between the baseline temperature $T_{0}$ and the pain tolerance temperature $T_{4}$ is reported in Table 3. The achieved results based on CNNs are also compared to the state-of-the-art results reported in previous works. At a glance, the designed deep learning architectures outperform the state-of-the-art results in every setting, except for the ECG modality. Regarding the aggregation of all physiological modalities, the second late fusion architecture performs best and sets a new state-of-the-art fusion performance with an average accuracy of 84.40%, which even outperforms the best fusion results reported in [41], where the authors could achieve an average classification performance of 83.1% by using both physiological and video features.

The deep architecture based on the EDA modality significantly outperforms all previously reported classification results with an average accuracy of 84.57%.

Based on these findings, further classification experiments were conducted, based on each physiological modality and also the best performing fusion architecture (Late Fusion (b)). The performance evaluation of the conducted experiments consisting of several binary classification experiments and a multi-class classification experiment is summarised in Table 4.

EDA significantly outperforms both EMG and ECG in all conducted classification experiments and constitutes the best performing single modality, which is consistent with the results reported in previous works. Both EMG and ECG depict similar classification performances and also perform poorly for almost all classification experiments. The discrimination between the baseline temperature $T_{0}$ and the pain threshold temperature $T_{1}$, as well as the two intermediate temperatures $T_{2}$ and $T_{3}$, constitute very difficult classification experiments that both modalities are unable to perform successfully. However, the classification performances of both modalities for the classification tasks $T_{0}$ vs. $T_{4}$ and $T_{1}$ vs. $T_{4}$ are significantly above chance level, which shows that higher temperatures of elicitation cause observable and measurable responses in the recorded physiological signals, that can be used to perform the classification tasks at a certain degree of satisfaction. However, the overall performance of the fusion architecture is greatly affected by the significantly poor performance of both ECG and EMG in comparison to EDA. As can be seen in Table 4, the EDA classification architecture outperforms the fusion architecture in almost all classification experiments (but not significantly), except for the classification task $T_{1}$ vs. $T_{4}$ and the multi-class classification task (the performance improvement of the fusion architecture is however not significant).

**Figure 5:**
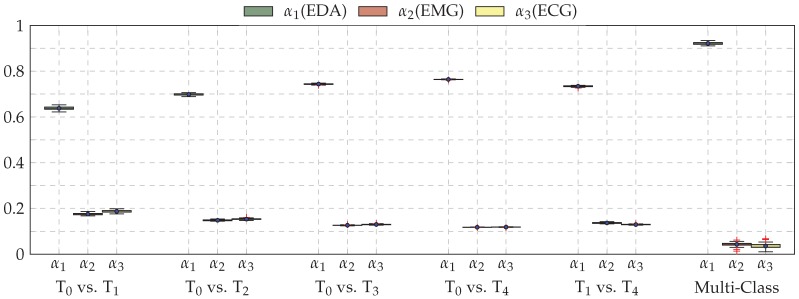
Box plots of the weighting parameters $\alpha_{1}$, $\alpha_{2}$ and $\alpha_{3}$ for the late fusion architecture (Late Fusion (b)), computed during the LOSO cross-validation evaluation of each conducted classification experiment. Within each box plot, the mean and median values of the performed LOSO cross-validation evaluation are depicted with a dot and a horizontal line, respectively.

The information stemming from both modalities EMG and ECG harms the optimisation process of the fusion architecture due to its inconsistency. However, it can be seen in Figure 5 that the fusion architecture is able to detect the sources of inconsistent information and dynamically adapt by systematically assigning higher weight values to EDA, while both ECG and EMG are assigned significantly lower weight values for all conducted classification tasks, and therefore improving the generalisation ability of the fusion architecture.

Subsequently, the performance of both EDA and late fusion architectures were further evaluated using different performance measures. In the case of binary classification experiments, *true positives*(*tp*) correspond to the number of correct acceptances, *false positives*(*fp*) correspond to the number of false acceptances, *true negatives*(*tn*) correspond to the number of correct rejections and *false negatives*(*fn*) correspond to the number of false rejections. These four values stem from the confusion matrix of an evaluated inference model and are used to define different performance measures. Those used for the current evaluation of the designed classification architectures are defined in Table 5.

**Figure 6:**
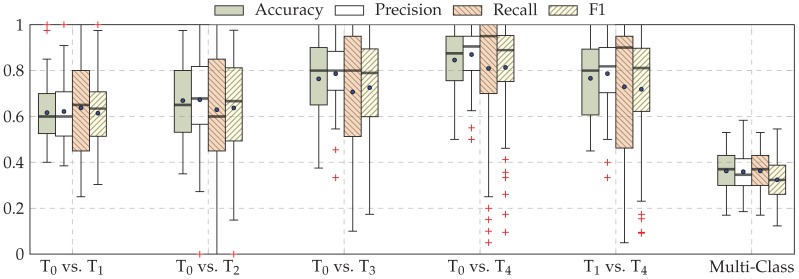
EDA classification performance. Within each box plot, the mean and median values of the respective performance evaluation metrics are depicted with a dot and a horizontal line, respectively.

**Figure 7:**
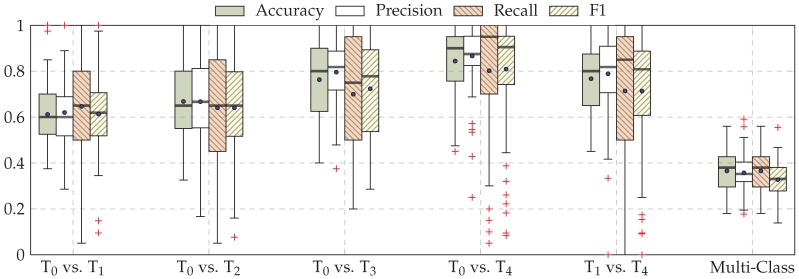
Late fusion classification performance (Late Fusion (b)). Within each box plot, the mean and median values of the respective performance evaluation metrics are depicted with a dot and a horizontal line, respectively.

**Figure 8:**
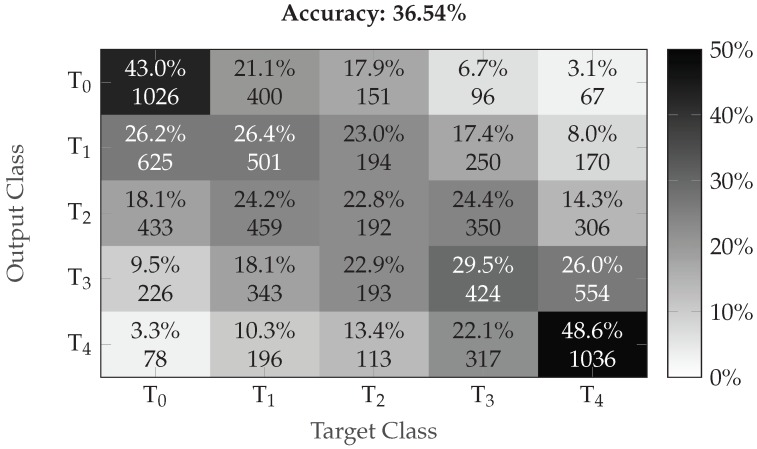
Multi-class classification performance (confusion matrix) of the fusion architecture (Late Fusion (b)). The darker the color the higher the corresponding performance.

The performance evaluation of the EDA architecture is depicted in Figure 6, while the performance evaluation of the fusion architecture is depicted in Figure 7. Considering binary classification experiments, both architectures are able to consistently discriminate between the baseline temperature $T_{0}$ and the other temperatures of pain elicitation. However, the performance of both architectures with regards to the five-class classification experiment suggests that the discrimination between all five levels of pain elicitation is a very challenging classification task. While the overall accuracy of each architecture is significantly above random performance (which is 20% in the case of a five-class classification task), the discrimination of the intermediate levels of pain elicitation remains very difficult, as can be seen in Figure 8. Both baseline and pain tolerance temperatures $T_{0}$ and $T_{4}$ can be classified with a relatively good performance. The classification performance of $T_{2}$ is barely above random performance and both $T_{1}$ and $T_{3}$ are mostly confused with $T_{0}$ and $T_{4}$, respectively. These results are however consistent with previous works on the same dataset.

We therefore compared the performance of the EDA and proposed late fusion approach to early works. For the sake of fairness, we considered the related works performed on the exact same dataset, using the exact same evaluation settings (LOSO with all 87 participants). The results depicted in Table 6 clearly show that the designed CNN architecture specific to EDA is able to consistently and significantly outperform previous approaches in all binary classification settings. Moreover, the authors of [56,57] reported overall accuracy performances of, respectively, 74.40% and 81.30% for the binary classification task $T_{0}$ vs. $T_{4}$ based uniquely on EDA. These approaches are also based on carefully designed hand-crafted features and are also significantly outperformed by the proposed CNN architecture specific to EDA.

Furthermore, we also compared the proposed late fusion approach with other fusion approaches proposed in early works. The results depicted in Table 7 show that the proposed fusion approach outperforms previous approaches for the binary classification task $T_{0}$ vs. $T_{4}$. Concerning the multi-class classification task, the proposed fusion approach also outperforms early approaches with an overall accuracy of 36.54%. The authors of [41] reported an overall accuracy of 33% with a classification model based on physiological modalities, while Werner et al. [58] reported an overall accuracy of 30.8% with a classification model based on head pose and facial activity descriptors.

Moreover, the designed fusion architecture was tested on the *BioVid Heat Pain Database (Part B)*. The database was generated using the same exact procedure as *Part A*. However, it consists of 86 participants and two additional EMG signals (from the corrugator and the zygomaticus muscles) were recorded. In this evaluation, we used the same signals as in *Part A* (EMG of the trapezius muscle, ECG and EDA), and used the same fusion architecture (Late fusion (b) depicted in Figure 4b). The computed results were subsequently compared with those of previous works. The corresponding results are depicted in Table 8.

The methods reported in previous works consist of fusion approaches involving all the recorded signals and based on hand-crafted features [37,56]. Although the fusion approach proposed in the current work (late fusion (b)) is based only on three of the recorded physiological signals, it is still able to outperform the previously proposed approaches, as depicted in Table 8. Therefore, it is believed that the performance of the architecture can be further improved by including the remaining signals (EMG corrugator, EMG zygomaticus, and Video) in the proposed architecture.

## 4. Discussion and Conclusions

This work explored the application of deep neural networks for pain intensity classification based on physiological data including ECG, EMG and EDA. Several CNN architectures, based on 1D and 2D convolutional layers, were designed and assessed based on the *BioVid Heat Pain Database (Part A)*. Furthermore, several deep fusion architectures were also proposed for the aggregation of relevant information stemming from all involved physiological modalities. The proposed architecture specific to EDA significantly outperformed the results presented in previous works in all classification settings. For the classification task $T_{0}$ vs. $T_{4}$, EDA achieved a state-of-the-art average accuracy of 84.57%. The proposed late fusion approach based on a weighted average of each modality specific model’s output also achieved state-of-the-art performances (average accuracy of 84.40% for the classification task $T_{0}$ vs. $T_{4}$), but was unable to significantly outperform the deep model based uniquely on EDA.

Moreover, all designed architectures were trained in an *end-to-end* manner. Therefore, it is believed that the pre-training and fine tuning at different levels of abstraction of the CNN architectures, as well as the combination with recurrent neural networks (in order to include the temporal aspect of the physiological signals in the inference model), could potentially improve the performance of the current system, since such approaches have been successfully applied in other domains of application such as facial expression recognition [59,60,61]. Finally, the recorded video data provide an additional channel that can be integrated into the fusion architecture in order to improve the performance of the whole system. Therefore, the video modality should also be evaluated and assessed in combination with the physiological modalities.

In summary, the performed assessment suggests that deep learning approaches are relevant for the inference of pain intensity based on 1D physiological data, and such methods are able to significantly outperform traditional approaches based on hand-crafted features. Domain expert knowledge could be bypassed by enabling the designed deep architecture to learn relevant features from the data. In the future iterations of the current work, approaches consisting of combining both learned and hand-crafted features should be addressed. In addition, the designed architectures should be also assessed by replacing the classification experiments by regression experiments. Additionally, several data transformation approaches applied to the recorded 1D physiological data in order to generate 2D visual representations (e.g., spectrograms) should also be investigated in combination with established deep neural network approaches, specifically designed for this type of data representation.

## Figures and Tables

**Table 1:** Deep classification architectures for each of the recorded physiological modality.

EDA				EMG & ECG			
Layer Name	No. Kernels (Units)	Kernel (Pool) Size	Stride	Layer Name	No. Kernels (Units)	Kernel (Pool) Size	Stride
Convolution	16	3	1	Convolution	16	11	1
Batch Normalisation	-	-	-	Batch Normalisation	-	-	-
Max Pooling	-	2	2	Max Pooling	-	2	2
Convolution	16	3	1	Dropout	-	-	-
Batch Normalisation	-	-	-	Convolution	16	11	1
Max Pooling	-	2	2	Batch Normalisation	-	-	-
Convolution	32	3	1	Max Pooling	-	2	2
Batch Normalisation	-	-	-	Dropout	-	-	-
Max Pooling	-	2	2	Convolution	32	11	1
Convolution	32	3	1	Batch Normalisation	-	-	-
Batch Normalisation	-	-	-	Max Pooling	-	2	2
Max Pooling	-	2	2	Dropout	-	-	-
Convolution	64	3	1	Convolution	32	11	1
Batch Normalisation	-	-	-	Batch Normalisation	-	-	-
Max Pooling	-	2	2	Max Pooling	-	2	2
Convolution	64	3	1	Dropout	-	-	-
Batch Normalisation	-	-	-	Convolution	64	11	1
Max Pooling	-	2	2	Batch Normalisation	-	-	-
Convolution	128	3	1	Max Pooling	-	2	2
Batch Normalisation	-	-	-	Dropout	-	-	-
Max Pooling	-	2	2	Convolution	64	11	1
Flatten	-	-	-	Batch Normalisation	-	-	-
Fully Connected	1024	-	-	Max Pooling	-	2	2
Dropout	-	-	-	Dropout	-	-	-
Fully Connected	512	-	-	Convolution	128	11	1
Dropout	-	-	-	Batch Normalisation	-	-	-
Fully Connected	c	-	-	Max Pooling	-	2	2
				Flatten	-	-	-
				Dropout	-	-	-
				Fully Connected	1024	-	-
				Dropout	-	-	-
				Fully Connected	512	-	-
				Dropout	-	-	-
				Fully Connected	c	-	-

ELU is used as activation function in both convolutional and fully connected layers, except for the last fully connected layer where a *softmax* activation function is used. The networks are further regularised by using *dropout* layers with a fixed dropout rate of 25%.

**Table 2:** Early fusion deep CNN architecture.

Layer Name	No. Kernels (Units)	Kernel (Pool) Size	Stride
Convolution	16	2×11	1×1
Convolution	16	2×11	1×1
Batch Normalisation	-	-	-
Max Pooling	-	1×2	1×2
Dropout	-	-	-
Convolution	32	1×11	1×1
Batch Normalisation	-	-	-
Max Pooling	-	1×2	1×2
Dropout	-	-	-
Convolution	32	1×11	1×1
Batch Normalisation	-	-	-
Max Pooling	-	1×2	1×2
Dropout	-	-	-
Convolution	64	1×11	1×1
Batch Normalisation	-	-	-
Max Pooling	-	1×2	1×2
Dropout	-	-	-
Convolution	64	1×11	1×1
Batch Normalisation	-	-	-
Max Pooling	-	1×2	1×2
Flatten	-	-	-
Dropout	-	-	-
Fully Connected	1024	-	-
Dropout	-	-	-
Fully Connected	512	-	-
Dropout	-	-	-
Fully Connected	c	-	-

The architecture is based on 2D convolutional layers. A 2D representation of the input data is generated by concatenating the three physiological modalities resulting in a tensor with the dimensionality 3×1152×1. Similar to the previous architectures (see Table 1), ELU is used as activation function in both convolutional and fully connected layers, except for the last fully connected layer where a *softmax* activation function is used. The network is further regularised by using *dropout* layers with a fixed dropout rate of 25%.

**Table 3:** Performance comparison to early work on the BVDB (Part A) for the classification task $T_{0}$ vs. $T_{4}$ in a LOSO cross-validation setting.

Method	ECG	EMG	EDA	Fusion
Werner et al. [36]	**62.00**	57.90	73.80	74.10
Kächele et al. [40,41]	53.90	58.51	81.10	82.73
Our Approaches (CNNs)	57.04±11.58	58.65±13.82	84.57±14.13	Early Fusion: 82.79±15.22 Late Fusion (a): 83.39±15.54 **Late Fusion (b):** 84.40±14.43

The performance metric consists of the average accuracy (in %) over the LOSO cross-validation evaluation (the standard deviation of the cross-validation results for the proposed approaches is also provided). The best performing approach for each modality and the aggregation of all modalities is depicted in bold.

**Table 4:** CNN Classification performance on the BVDB (Part A) in a LOSO cross-validation setting (the multi-class classification task corresponds to the five-class classification task $T_{0}$ vs. $T_{1}$ vs. $T_{2}$ vs. $T_{3}$ vs. $T_{4}$).

Task	ECG	EMG	EDA	Late Fusion (b)
$T_{0}$ vs. $T_{1}$	49.71±06.90	49.71±02.77	61.67±12.54 ^†^	61.15±12.22 ^a,b^
$T_{0}$ vs. $T_{2}$	50.72±07.30	50.29±03.60	66.93±16.19 ^†^	66.81±15.92 ^a,b^
$T_{0}$ vs. $T_{3}$	52.87±09.32	53.25±08.93	76.38±14.70 ^†^	76.29±14.62 ^a,b^
$T_{0}$ vs. $T_{4}$	57.04±11.58	58.65±13.82	84.57±14.13 ^†^	84.40±14.43 ^a,b^
$T_{1}$ vs. $T_{4}$	58.07±12.36	58.79±12.08	76.61±15.38	76.72±15.02 ^a,b,†^
*Multi-Class*	23.23±05.62	22.85±05.65	36.25±09.01	36.54±08.55 ^a,b,†^

The performance metric consists of the average accuracy (in %) over the LOSO cross-validation evaluation (the standard deviation of the cross-validation results is also provided). The best performing approach for each classification task is depicted in bold. We also performed a significance test between the fusion approach and each single modality, using a Wilcoxon signed rank test with a significance level of 5%: (^a^) indicates a significant performance improvement between EMG and the fusion approach; (^b^) indicates a significant performance improvement between ECG and the fusion approach; and (^†^) indicates no significant improvement between EDA and the fusion approach.

**Table 5:** Classification performance measures.

Measure	Binary Classification	Multi-Class Classification
Accuracy	$\frac{tp+tn}{tp+tn+fp+fn}$	$\frac{1}{c}\sum_{i=1}^{c}\frac{tp_{i}+tn_{i}}{tp_{i}+tn_{i}+fp_{i}+fn_{i}}$
Precision	$\frac{tp}{tp+fp}$	$\frac{1}{c}\sum_{i=1}^{c}\frac{tp_{i}}{tp_{i}+fp_{i}}$
Recall	$\frac{tp}{tp+fn}$	$\frac{1}{c}\sum_{i=1}^{c}\frac{tp_{i}}{tp_{i}+fn_{i}}$
F1 score	$\frac{2\times Precision\times Recall}{Precison+Recall}$	

In the case of multi-class classification experiments: $tp_{i}$ corresponds to true positives, $tn_{i}$ corresponds to true negatives, $fp_{i}$ corresponds to false positives and $fn_{i}$ corresponds to false negatives in the confusion matrix associated with the *i*th class. Furthermore, since the dataset used for the evaluation of the performance of the designed architectures is balanced, we use the macro-averaged F1 score in the case of multi-class classification.

**Table 6:** EDA performance comparison to early work on the BVDB (Part A) in a LOSO cross-validation setting.

Method	$T_{0}$ vs. $T_{1}$	$T_{0}$ vs. $T_{2}$	$T_{0}$ vs. $T_{3}$	$T_{0}$ vs. $T_{4}$
Werner et al. [36]	55.40	60.20	65.90	73.80
Lopez-Martinez et al. [55]	56.44	59.40	66.00	74.21
**Our Approach (CNN)**	61.67±12.54	66.93±16.19	76.38±14.70	84.57±14.13

The performance metric consists of the average accuracy (in %) over the LOSO cross-validation evaluation. The best performing approach for each classification task is depicted in bold.

**Table 7:** Fusion performance comparison to early work on the BVDB (Part A) in a LOSO cross-validation setting for the classification task $T_{0}$ vs. $T_{4}$.

Approach	Description	Performance
Werner et al. [58]	Early Fusion with Random Forests (Head Pose and Facial Activity Descriptors)	72.40
Werner et al. [36]	Early Fusion with Random Forests (EDA, EMG, ECG, Video)	77.80
Kächele et al. [56]	Early Fusion with Random Forests (EDA, ECG, Video)	78.90
Kächele et al. [57]	Late Fusion with Random Forests and Pseudo-inverse (EDA, EMG, ECG, Video)	83.10
**Our Approach (CNN)**	**Late Fusion (b) with CNNs (EDA, EMG, ECG)**	84.40±14.43

The performance metric consists of the average accuracy (in %) over the LOSO cross-validation evaluation. The best performing approach is depicted in bold.

**Table 8:** Fusion performance comparison to early work on the BVDB (Part B) in a LOSO cross-validation setting for the classification task $T_{0}$ vs. $T_{4}$.

Approach	Description	Performance
Kächele et al. [56]	Late Fusion with SVMs and Mean Aggregation (EMG (zygomaticus), EMG (corrugator), EMG (trapezius), ECG, EDA, Video)	76.60
Walter et al. [37]	Early Fusion with SVM (EMG (zygomaticus), EMG (corrugator), EMG (trapezius), ECG, EDA)	77.05
**Our Approach (CNN)**	**Late Fusion (b) with CNNs (EMG (trapezius), ECG, EDA)**	79.48±14.96

The performance metric consists of the average accuracy (in %) over the LOSO cross-validation evaluation. The best performing approach is depicted in bold.
